# Supplementary material for: Updating the Methodology of Identifying Maize Hybrids Resistant to Ear Rot Pathogens and Their Toxins—Artificial Inoculation Tests for Kernel Resistance to Fusarium graminearum, F. verticillioides, and Aspergillus flavus
Source: J Fungi (Basel). 2022 Mar 11;8(3):293. doi: 10.3390/jof8030293 (PMC8954121; doi:10.3390/jof8030293)
Supplement: Supplementary file 1 [file jof-08-00293-s001.zip › jof 1597279 Supplementary Tables to print.pdf]

Table S1. Kernel resistance tests of maize hybrids to toxigenic fungi. ANOVA of the ear rot data of maize resistance test to toxigenic fungi (2017–2018).

Source of variance	SS	df	MS	F
Hybrid A	1381.31	17	81.25	3.50**
Tox. spp. B	34725.54	3	11575.18	498.93***
Isolate C	603.00	1	603.00	25.99***
Year D	3749.36	1	3749.36	161.61***
A × B	3716.68	51	72.88	3.14***
A × C	577.27	17	33.96	1.46
A × D	968.62	17	56.98	2.46
B × C	1847.21	3	615.74	26.54***
B × D	11770.75	3	3923.58	169.12***
C × D	388.63	1	388.63	16.75***
A × B × C	1752.40	51	34.36	1.48*
A × B × D	2878.55	51	56.44	2.43***
A × C × D	319.94	17	18.82	0.81ns
B × C × D	1048.26	3	349.42	15.06***
A × B × C × D	989.37	51	19.40	0.84ns
Within	13387.95	576	23.24	
Total	80104.82	863		

*** P = 0.001, ** P = 0.01, * P = 0.05, ns = not significant

Table S2. Kernel resistance test of maize hybrids to toxigenic fungi. Mycotoxin contamination of maize ears following *F. graminearum*, *F. verticillioides*, and *A. flavus* inoculation in maize hybrids with two isolates per year, 2017–2018.

Hybrid	DON mg/kg				Varian-ce	Fumonisin B_1+B_2, mg/kg					Varian-ce	Aflatoxin B1					Variance
	Fg3 17	Fg3 18	Fg4 18	Mean		Fv1 17	Fv2 17	Fv1 18	Fv2 18	Mean		Af2 17	Af1 17	AF2 18	AF1 18	Mean	
**P 9537**	**7.3**	**6.4**	**8.1**	**7.27**	**14**	**7.0**	**3.6**	**1.4**	**0.3**	**3.1**	**8.7**	**48**	**30**	**5**	**4**	**22**	**451**
**DKC 4590**	**4.6**	**2.3**	**19.4**	**8.75**	**76**	**5.3**	**2.2**	**0.9**	**0.9**	**2.3**	**4.4**	**36**	**18**	**17**	**15**	**22**	**95**
Fornad	**15.7**	**3.2**	**13.6**	**10.84**	**58**	7.2	10.1	0.0	0.6	4.5	24.5	145	162	81	161	137	1467
4517	**15.4**	**11.4**	**7.9**	**11.58**	**37**	15.1	21.9	2.3	6.6	11.4	76.6	119	144	31	17	78	3989
**DKC 4717**	**13.4**	**15.4**	**12.7**	**13.81**	**48**	**2.2**	**2.0**	**0.3**	**0.0**	**1.1**	**1.2**	**41**	**58**	**38**	**24**	**40**	**195**
**DKC 4943**	**14.7**	**7.0**	**20.2**	**13.98**	**76**	**1.4**	**1.9**	**0.3**	**0.1**	**0.9**	**0.7**	**14**	**65**	**73**	**25**	**44**	**848**
Szegedi 521	**16.1**	**17.8**	**10.5**	**14.80**	**51**	**2.9**	**2.7**	**3.7**	**1.6**	**2.7**	**0.8**	516	334	45	264	290	37904
P 9903	3.9	25.0	22.2	17.04	104	**2.0**	**3.2**	**1.3**	**0.2**	**1.7**	**1.5**	**63**	**14**	**64**	**13**	**39**	**834**
**Cardixxio Duo**	**8.8**	**29.8**	**13.4**	17.32	**157**	**1.3**	**3.0**	**0.5**	**0.1**	**1.2**	**1.6**	**28**	**69**	**20**	**1**	**30**	**822**
PR37F80	**25.2**	**11.6**	**18.9**	18.57	**116**	18.7	9.1	2.4	0.5	7.7	67.5	89	84	40	328	135	16997
DKC 4541	6.3	18.4	31.9	18.85	197	4.5	5.7	1.4	1.0	3.2	5.3	64	118	14	34	58	2049
DKC 5542	16.3	9.6	32.6	19.54	189	**2.2**	**1.6**	**0.5**	**0.9**	**1.3**	**0.6**	333	697	321	3	339	80473
P 9241	6.0	25.9	26.7	19.57	187	**3.2**	**1.6**	**1.2**	**1.2**	**1.8**	**0.8**	**21**	**12**	**9**	**37**	**20**	**158**
P 9911	21.9	14.0	23.5	19.79	115	7.8	3.2	6.2	8.7	6.5	5.7	102	102	31	2	59	2577
DKC 5830	14.3	36.9	17.0	22.72	231	**1.0**	**2.0**	**0.6**	**0.3**	**1.0**	**0.6**	847	77	356	8	322	145134
Siló Star	23.0	16.6	30.9	23.47	172	**2.4**	**0.6**	**0.9**	**0.3**	**1.1**	**0.9**	350	81	64	314	202	22711
Korimbos	20.7	16.9	39.8	25.79	266	**1.0**	**0.9**	**0.6**	**0.7**	**0.8**	**0.0**	345	779	70	1	299	124587
Valkür	99.5	43.5	46.8	63.28	1659	**2.8**	**1.1**	**2.3**	**0.3**	**1.6**	**1.3**	**15**	**16**	**0**	**21**	**13**	**82**
Mean	18.5	17.3	22.0	19.28	208	4.9	4.2	1.5	1.3	3.0	11.3	176	159	71	71	119	24521
LSD 5%				21.5						4.0						66	

Correlations	Fg3	Fg3	Fg4	Mean	Corr.	Fv1 17	Fv2 17	Fv1 18	Fv2 18	Corr.	Af2 17	Af1 17	AF2 18	AF1 18
					Fv2 17	0.77***				Af1 17	0.40			
Fg3	0.51*				Fv1 18	0.37	0.13			AF2 18	0.74***	0.41		
Fg4	0.58*	0.40			Fv2 18	0.44	0.49*	0.77***		AF1 18	0.21	-0.05	-0.12	
Mean	0.91***	0.74***	0.77***		Mean	0.91***	0.89***	0.51*	0.71***	Átlag	0.88***	0.74***	0.74***	0.29

*** P=0.001, *P = 0.05 Fg = *F. graminearum*, Fv = *F. verticillioides*, Af = *A. flavus*

Table S3. Kernel resistance test of maize hybrids. Correlations counted from the data from the 2017–2018 maize ear rot resistance tests are from Table 4.

Trait	Toxigenic spp., ear rot %			Toxins			Control				
	**Fg**	**Fv**	**Af**	**DON mg/kg**	**FB_1+B_2 mg/kg**	**AFB1 µg/kg**	**F. Ear rot %**	**Af ear rot%**	**DON mg/kg**	**FB_1+B_2 mg/kg**	**AFB1 µg/kg**
Fg											
Fv	0.319										
Af	0.512*	0.575*									
DON	0.419	-0.331	-0.359								
FB_1+B_2	0.209	0.59***	0.178	-0.226							
AFB_1	-0.172	-0.171	-0.137	0.014	-0.168						
F. Ear rot	0.123	0.827***	0.491*	-0.575***	0.637**	-0.141					
Afl ear rot	0.152	0.236	0.070	-0.165	0.271	-0.201	0.428				
DON	-0.261	-0.185	-0.296	0.259	-0.271	0.351	-0.372	-0.046			
FB_1+B_2	-0.191	0.418	-0.141	-0.312	0.715***	-0.117	0.486*	0.047	-0.259		
AFB_1	-0.520*	-0.162	-0.229	-0.129	0.052	0.509*	0.010	-0.205	0.100	0.098	
FAO No.	-0.071	0.366	0.453	-0.753***	0.062	-0.478*	0.505*	0.232	-0.342	0.043	-0.267

*** P = 0.001; *** P = 0.01; ** P = 0.02; P = 0.05; [x]Fg = *F. graminearum*; Fv = *F. verticillioides*; Af = *A. flavus*.

Table S4. Kernel resistance tests of maize hybrids to toxigenic fungi. Reductions in the ear rot severity and toxin contamination are compared to the most susceptible hybrid in the given column. Data are shown as percentages (Szeged, 2017–2018).

Hybrid	Toxic spp., ear rot %			Toxins			Control					Mean	FAO No.
	Fg*	Fv	Af	DON mg/kg	FB_1+B_2 mg/kg	AFB1 µg/kg	F. Ear rot %	Afl ear rot %	DON mg/kg	FB1+B2 mg/kg	AFB1 µg/kg		
Szegedi 521	53.4	78.8	79.1	69.1	76.4	94.2	66.6	100.0	100.0	89.9	84.5	82.3	350
Korimbos	55.6	72.6	69.8	88.5	79.9	93.6	63.3	100.0	100.0	78.3	87.8	79.9	370
DKC 4590	47.0	80.8	39.3	72.6	92.9	91.3	61.9	100.0	92.5	97.4	97.2	79.8	470
P9537	41.7	73.3	56.8	73.1	73.3	88.6	36.1	100.0	95.8	94.2	97.2	76.9	390
4517	57.3	69.2	68.6	86.2	90.3	93.6	26.3	70.6	92.1	60.9	98.2	73.9	360
P9241	49.3	61.6	49.3	78.5	84.4	88.1	49.5	100.0	97.9	72.8	67.4	72.3	390
DKC 4717	67.6	96.8	89.8	59.2	89.3	11.7	82.7	100.0	0.0	94.0	69.6	70.0	575
Cardixxio Duo	22.2	76.9	37.9	64.1	88.8	4.9	69.6	100.0	100.0	98.7	97.2	69.9	560
DKC 5542	16.7	87.8	94.2	0.0	0.0	96.2	96.1	100.0	74.6	92.8	94.5	68.4	730
Fornad	42.6	77.7	66.8	62.9	90.8	40.3	70.2	100.0	45.8	95.6	57.0	68.2	490
Siló Star	41.9	73.0	17.0	77.9	61.0	86.9	53.2	70.6	86.7	93.9	76.2	66.5	400
DKC 4943	46.4	73.1	79.3	69.1	91.8	0.0	45.9	100.0	100.0	61.6	63.5	66.4	540
P9903	79.0	83.1	76.0	76.6	85.3	14.4	38.5	94.1	93.8	90.8	0.0	65.8	560
P9911	26.8	74.5	72.8	68.7	43.5	82.5	28.0	0.0	100.0	77.3	88.4	59.8	450
DKC 4541	24.5	5.3	25.4	70.2	72.2	83.0	0.0	76.5	97.9	81.6	90.6	58.0	370
DKC 5830	0.0	27.5	0.0	70.7	85.7	60.0	14.9	100.0	100.0	87.1	79.0	55.8	420
Valkür	53.9	26.0	65.3	81.7	33.1	77.0	12.2	100.0	100.0	0.0	64.6	54.7	520
PR37F80	45.3	0.0	39.6	82.9	91.5	59.5	8.0	47.1	56.3	78.0	92.8	53.4	420
Mean	45.4	67.0	60.4	73.6	78.2	68.6	48.4	91.7	90.2	85.0	82.7	67.9	

*Fg = *F. graminearum*; Fv = *F. verticillioides*; Af = *A. flavus*; DON = deoxynivalenol; FB_1+B_2 = fumonisin B_1+B_2; AFB1 = aflatoxin B_1; F. Control = natural fusarium infection in the check; Asp. Check = *A. flavus* natural infection in their control; FAO No. = earliness or lateness of the hybrids according to the FAO international methodology.

Table S5. Kernel resistance of maize hybrids to toxigenic fungi, ANOVA of the ear rot data from Table 5, 2019–2020.

Source of var.	SS	df	MQ	F	LSD 5%
Genotype A	3403.7	17	200.22	6.89***	2.16
Tox. spp. B	74832.8	3	24944.26	858.96***	1.02
Isolate C	38.3	1	38.32	1.32	
Year D	6143.3	1	6143.26	211.54***	0.72
A × B	10170.7	51	199.43	6.86***	
A × C	426.5	17	25.09	0.86	
A × D	2081.5	17	122.44	4.21***	
B × C	80.1	3	26.68	0.92	
B × D	18478.6	3	6159.54	212.10***	
C × D	29.2	1	29.18	1.00	
A × B × C	1283.9	51	25.17	0.87	
A × B × D	6310.4	51	123.73	4.26***	
A × C × D	531.2	17	31.25	1.08	
B × C × D	101.0	3	33.65	1.16	
A × B × C × D	1651.3	51	32.38	1.11	
Within	16729.8	576	29.04		
Total	142292.1	863			

*** P = 0.001.

Table S6. Kernel resistance of maize hybrids to toxigenic fungi. Mycotoxin contamination of maize ears following *F. graminearum, F. verticillioides,* and *A. flavus* inoculation with two isolates per year, 2019–2020.

**Hybrid**	**DON mg/kg**					**Varian-ce**	**Fumonisin B1+B2, mg/kg**					Variance	**Aflatoxin B1 μg/kg**					Variance
	**Fg3 19**	**Fg4 19**	**Fg3 20**	**Fg4 20**	**Mean**		**Fv1 19**	**Fv2 19**	**Fv1 20**	**Fv2 20**	**Mean**	Variance	**Af 19**	**Af2 19**	**Afl1**	**Afl2**	**Mean**	
**Sy Talisman**	**3.9**	**9.6**	**6.1**	**10.3**	**7.5**	9	**1.0**	**0.8**	**4.9**	**0.5**	**1.8**	**4.2**	**135**	**13**	**42**	**92**	**71**	**2914**
Kathedralis	**15.4**	**23.7**	**0.0**	**14.5**	**13.4**	97	0.5	1.5	10.3	2.7	3.8	20.0	40	32	805	567	361	150285
Konfites	**25.2**	**21.0**	**4.6**	**21.8**	**18.1**	85	1.0	0.6	12.0	0.3	3.5	32.6	**43**	**41**	**69**	**59**	**53**	**179**
**Armagnac**	**18.1**	**45.6**	**7.2**	**30.2**	**25.3**	272	**1.0**	**0.0**	**0.3**	**2.2**	**0.9**	**0.9**	**150**	**58**	**148**	**35**	**98**	**3591**
**P9718E**	**41.6**	**49.6**	**11.6**	**10.6**	**28.4**	408	**1.9**	**1.2**	**3.3**	**0.7**	**1.8**	**1.4**	**108**	**356**	**21**	**155**	**160**	**20155**
P0725	**3.3**	**41.7**	**44.0**	**25.3**	**28.6**	354	3.9	0.0	7.3	5.8	4.1	10.1	1	1	1354	1096	613	510486
**Koregraf**	**54.5**	**0.3**	**22.5**	**45.6**	**30.7**	594	**1.4**	**1.2**	**6.4**	**0.9**	**2.5**	**6.9**	**40**	**42**	**59**	**6**	**37**	**493**
ES Harmonium	116.6	26.2	3.4	4.2	37.6	2885	**1.4**	**2.3**	**4.0**	**0.7**	**2.1**	**2.1**	**162**	**33**	**28**	**25**	**62**	**4455**
ES Lagoon	**17.1**	**29.6**	**3.4**	**105.3**	**38.9**	**2076**	0.8	3.2	6.3	2.2	3.1	5.5	143	16	3261	164	896	2490153
Illango	**15.1**	**67.6**	**104.7**	**0.7**	**47.0**	**2306**	**0.9**	**5.2**	**2.8**	**1.6**	**2.6**	**3.5**	56	85	56	4076	1068	4020880
DKC 5830	50.7	111.4	24.2	8.8	48.8	**2044**	0.9	5.2	4.6	2.3	3.3	4.0	**56**	**85**	**56**	**784**	**245**	**129188**
DKC 4541	13.2	149.5	24.0	9.9	49.1	4509	2.9	3.2	8.3	1.6	4.0	8.9	**465**	**134**	**79**	**19**	**174**	**39777**
P9415	38.1	129.4	17.5	86.9	68.0	**1301**	**0.5**	**0.9**	**3.6**	**0.6**	**1.4**	**2.2**	**50**	**83**	**6**	**11**	**38**	**1307**
Kleopatras	154.9	74.3	6.2	61.5	74.2	3768	**0.9**	**2.3**	**2.1**	**2.0**	**1.8**	**0.4**	1	12	37	1190	310	344405
Korimbos	55.9	49.9	48.5	150.0	76.1	2439	0.8	0.3	16.1	5.4	5.7	54.1	**11**	**47**	**6**	**111**	**44**	**2344**
Valkür	87.1	62.1	24.3	157.0	82.6	3125	**1.6**	**0.5**	**2.1**	**2.0**	**1.6**	**0.5**	**0**	**20**	**487**	**314**	**205**	**55885**
Sy Zoan	120.7	90.8	168.2	5.7	96.3	4665	2.6	1.2	7.5	15.3	6.7	40.6	2	15	69	4947	1258	6048342
SY Zephir	184.4	193.7	20.6	5.4	101.0	10388	**2.7**	**0.8**	**2.1**	**0.6**	**1.5**	**1.0**	149	6	2377	20	638	1348197
Mean	56.4	65.3	30.0	41.9	48.4	2364	1.5	1.7	5.8	2.6	2.9	11.0	90	60	498	760	352	842946
LSD 5%					ns						ns						ns	

Correlation	Fg3	Fg4	Fg3	Fg4
Fg4	0.44			
Fg3	0.15	0.19		
Fg4	0.03	-0.11	-0.14	
Mean	0.75***	0.69**	0.47	0.35

Corr.	Fv1 19	Fv2 19	Fv1 20	Fv2 20
Fv2 19	-0.22			
Fv1 20	-0.08	0.04		
Fv2 20	-0.02	0.05	0.26	
Mean	0.34	0.43	0.61*	0.64*

Corr.	Afl1	Afl2	Afl1	Afl2	Mean
Afl2	0.27				
Afl1	0.08	-0.30			
Afl2	-0.31	-0.11	-0.15		
Mean	-0.16	-0.21	0.45	0.81***	

**** P = 0.001, * P = 0.05. **Bold numbers**: low toxin contamination and stable toxin performance in the three toxins measured. **Bold hybrid name**: low toxin contamination and variance (high stability) We have Talisman with mean 71, and variance 2914.

Table S7. Kernel resistance tests of maize hybrids to toxigenic fungi. Correlation coefficients between traits in Table 6 (Szeged, 2019–2020).

Correlations from Table 10	Fg	Fv	Af	DON	FB_1+B_2	AFB1	F. check.	Af. check.	DON	FB_1+B_2	AFB1
Fv	0.110										
Af	-0.161	0.743***									
DON	0.482*	-0.112	-0.364								
FB_1+B_2	-0.055	0.202	0.154	0.225							
AFB1	0.273	0.005	0.000	0.389	0.410						
F. check.	-0.091	0.619**	0.539*	-0.496*	0.059	-0.052					
Af. check.	0.282	0.190	-0.021	0.049	0.320	0.706**	0.143				
DON	-0.185	0.319	0.178	0.033	0.201	-0.475*	0.006	-0.113			
FB_1+B_2	0.249	0.396	0.475*	0.005	-0.006	-0.052	0.268	-0.023	0.314		
AFB1	-0.227	-0.074	-0.077	-0.044	0.179	0.375	-0.175	0.408	-0.185	-0.437	
FAO	0.269	-0.342	-0.367	0.482*	0.258	0.286	-0.601*	0.101	0.077	0.022	0.254

*** P = 0.001; ** P = 0.01; * P = 0.05. [x]Fg = *F. graminearum*; Fv = *F. verticillioides*; Af = *A. flavus.*

Table S8. Kernel resistance tests of maize hybrids to toxigenic fungi. Reductions in the disease severity and toxin contamination compared to the most susceptible hybrid in the given column in Table 5 (Szeged, 2019–2020).

Hybrid	Ear Rot Severity %			Toxin Content			Ear Rot Check		Toxin Content Control			Mean	Variance	FAO No.
	Fg %	Fv %	Afl %	DON mg/kg	FB1+B2 mg/kg	AFB1 ppb	F. Check	Af. Check	DON mg/kg	FB1+B2 mg/kg	AFB1 mg/kg			
ES Harmonium	67.5	85.8	74.2	62.8	68.5	95.1	18.0	100.0	96.5	85.7	99.7	77.6	576	380
Koregraf	53.0	73.7	58.5	69.6	63.0	97.1	68.4	100.0	100.0	94.8	69.2	77.0	309	410
Sy Talisman	61.8	75.0	53.1	92.6	72.9	94.4	10.8	100.0	79.9	90.4	99.8	75.5	697	250
Valkür	18.6	93.9	91.4	18.2	76.8	83.7	82.0	100.0	64.5	85.7	100.0	74.1	863	731
Armagnac	44.0	85.9	81.1	75.0	86.9	92.2	55.7	40.0	35.2	71.9	99.2	69.7	503	490
Konfites	71.3	62.0	40.1	82.0	47.6	95.8	31.3	100.0	52.8	66.7	99.8	68.1	583	430
P9718E	38.2	69.7	57.3	0.0	77.2	49.3	70.6	100.0	95.4	79.3	99.7	67.0	897	390
P9415	9.4	63.0	52.7	51.4	79.2	97.0	21.8	100.0	83.8	58.9	100.0	65.2	937	350
Kleopatras	1.0	72.8	82.7	26.5	72.9	75.4	46.5	100.0	90.3	59.1	90.1	65.2	899	630
SY Zephir	4.1	76.8	66.8	71.9	73.7	87.3	47.3	40.0	82.5	58.2	99.7	64.4	696	390
Korimbos	59.4	91.4	80.2	24.7	14.8	96.5	75.1	100.0	2.9	94.8	64.3	64.0	1216	575
Kathedralis	37.5	58.2	0.0	86.8	43.5	71.3	29.9	100.0	92.6	69.3	100.0	62.6	1041	490
P0725	55.0	72.4	80.6	71.7	38.2	51.3	42.1	40.0	100.0	97.9	30.5	61.8	588	560
ES Lagoon	44.1	80.1	58.0	61.5	53.2	28.8	37.1	40.0	100.0	60.5	98.3	60.1	567	460
DKC 5830	51.7	67.6	25.1	53.5	51.2	80.5	45.1	100.0	39.4	0.0	100.0	55.8	914	560
Illango	43.6	67.0	37.8	53.7	60.6	15.1	41.5	0.0	98.7	86.8	0.0	45.9	1044	530
Sy Zoan	0.0	71.2	72.9	4.6	0.0	0.0	41.0	0.0	100.0	70.7	100.0	41.8	1782	560
DKC 4541	30.5	0.0	7.0	51.7	40.0	86.1	0.0	40.0	0.0	45.6	100.0	36.4	1174	370
Mean	38.4	74.5	60.0	56.4	60.0	76.3	44.9	76.5	77.3	75.1	91.2	66.6	899	503

Fg = *F. graminearum*; Fv = *F. verticillioides*; Afl = *A. flavus*; DON = deoxynivalenol; FB = fumonisin B_1+B_2; AFB1 = aflatoxin B_1, F; Check = natural fusarium infection in the control; Af. Check = *A. flavus* natural infection in the control; FAO No. = earliness or lateness of the hybrids according to the FAO international methodology.

Table S9. Kernel resistance tests of maize hybrids to toxigenic fungi. Toxin contamination for one percentage of visual infection in maize resistance test against *F. graminearum*, *F. verticillioides*, and *A. flavus*, 2017-2018, and 2019–2020.

**Hybrid 2017/2018**	Rates between toxin (mg/kg) and ear rot%, mg/kg			Mean	Varian-ce	**Hybrid 2019/2020**	Rates between toxin (mg/kg) and ear rot%, mg/kg			Mean	Varian-ce
	DON/ GER	$FB_{1,2}$/ FER	AFB1/ AER				DON/ GER	$FB_{1,2}$/ FER	AFB1/ AER		
**DKC 4541**	0.95	2.84	0.162	1.32	1.9	DKC 4541	1.98	2.48	2.967	2.48	0.2
DKC 5830	1.11	3.59	1.092	1.93	2.1	DKC 5830	2.75	6.25	4.100	4.37	3.1
Korimbos	3.04	21.53	6.181	10.25	97.9	Korimbos	5.30	40.50	7.863	17.89	385.1
Valkür	2.90	11.39	0.473	4.92	32.9	Valkür	2.87	15.50	0.136	6.17	67.2
P 9537	0.62	9.44	0.152	3.41	27.4	**Sy Talisman**	0.55	4.50	0.151	1.74	5.8
PR37F80	0.71	8.95	0.285	3.31	23.9	**Konfites**	0.61	5.61	0.413	2.21	8.7
**Fornad**	0.76	3.79	0.479	1.67	3.4	**SY Zephir**	0.84	4.73	0.263	1.94	5.9
**DKC 4590**	0.78	6.34	0.144	2.42	11.6	**Armagnac**	1.28	3.78	0.367	1.81	3.1
**DKC 4943**	0.92	2.94	0.112	1.32	2.1	P9415	1.53	2.30	5.573	3.14	4.6
4517	0.96	13.10	0.471	4.84	51.2	**Konfites**	1.79	5.72	0.154	2.55	8.2
P 9911	1.03	21.49	0.459	7.66	143.5	P0725	1.80	9.16	0.557	3.84	21.6
**DKC 4717**	1.04	2.44	0.167	1.21	1.3	Koregraf	1.85	5.88	3.733	3.82	4.1
**P 9903**	1.12	5.34	0.188	2.21	7.5	ES Lagoon	1.97	9.72	0.891	4.19	23.2
**Cardixxio Duo**	1.25	5.38	0.102	2.24	7.7	Kleopatras	2.12	4.11	2.552	2.93	1.1
DKC 5542	1.39	4.05	3.442	2.96	1.9	Illango	2.34	4.94	0.622	2.64	4.7
Siló Star	1.56	4.02	1.284	2.29	2.3	Sy Zoan	2.72	14.17	0.322	5.74	54.8
P 9241	1.60	7.15	0.199	2.98	13.5	ES Harmonium	3.27	9.13	0.842	4.41	18.2
Szegedi 521	2.69	13.51	2.538	6.25	39.6	P9718E	4.62	3.10	3.100	3.61	0.8
**Mean**	**1.36**	**8.18**	**0.996**	**3.20**	**24.1**	**Mean**	**2.23**	**8.42**	**1.92**	**4.19**	**34.5**
Max/Min*	4.86	8.83	60.59			Max/Min	9.64	17.61	57.93		
Risk group	Low	Low to medium		Medium to high			High				

Bold: Hybrid names with low toxin production as a percentage of visual infection, good resistance, and high stability to all toxigenic fungi.*Rate between the maximum and minimum values in the column
